# Experimental study of dexamethasone-loaded hollow hydroxyapatite microspheres applied to direct pulp capping of rat molars

**DOI:** 10.3389/fendo.2023.1192420

**Published:** 2023-08-03

**Authors:** Xiaoli Liu, Yuandong Xie, Weijia Gao, Luoning Zhan, Ling Hu, Linjing Zuo, Yi Li

**Affiliations:** ^1^ Jilin Provincial Key Laboratory of Tooth Development and Bone Remodeling, Department of Pediatric Dentistry, Hospital of Stomatology, Jilin University, Changchun, Jilin, China; ^2^ Jilin Provincial Key Laboratory of Tooth Development and Bone Remodeling, Department of Dental Implantology, Hospital of Stomatology, Jilin University, Changchun, Jilin, China

**Keywords:** hollow hydroxyapatite, dexamethasone, live pulp preservation, reparative dentin, immunohistochemical staining (IHC)

## Abstract

**Background:**

Dexamethasone (DEX) exerts anti-inflammatory and osteogenic effects. Hydroxyapatite is commonly used in bone repair due to its osteoconductivity, osseointegration, and osteogenesis induction. Hollow hydroxyapatite (HHAM) is often used as a drug carrier.

**Objective:**

This study aimed to investigate the histological responses of exposed dental pulp when dexamethasone-loaded nanohydroxyapatite microspheres (DHHAM) were used as a direct capping agent.

**Methods:**

Cavities were created in the left maxillary first molar of Wistar rats and filled with Dycal, HHAM, and DHHAM. No drug was administered to the control group. The rats were sacrificed at 1, 2, and 4 weeks after the procedure. The molars were extracted for fixation, demineralization, dehydration, embedding, and sectioning. H&E staining was performed to detect the formation of reparative dentin. H&E and CD45 immunohistochemical staining were performed to detect pulp inflammation. Immunohistochemical staining was performed to assess the expressions of dentin matrix protein 1 (DMP-1), interleukin (IL)-6, tumor necrosis factor (TNF)-α, and IL-1β.

**Results:**

The results of H&E and CD45 immunohistochemical staining showed that the degree of inflammation in the DHHAM group was less than that in the Control and HHAM groups at 1, 2, and 4 weeks after capping of the rat molar teeth (p<0.01). The H&E staining showed that the percentage of reparative dentin formed in the DHHAM group was higher than that in the Control, HHAM (p<0.001), and Dycal groups (p<0.01) at 1 and 2 weeks, and was significantly higher than that in the Control group (p<0.001) and the HHAM group (p<0.01) at 4 weeks. The immunohistochemical staining showed a lower range and intensity of expression of IL-1β, IL-6, and TNF-α and high expression levels of DMP-1 in the DHHAM group at 1, 2, and 4 weeks after pulp capping relative to the Control group.

**Conclusions:**

DHHAM significantly inhibited the progression of inflammation and promoted reparative dentin formation.

## 1 Introduction

Exposure of a tooth, whether through mechanical means or trauma, increases the likelihood of pulp infection. If left untreated, this condition can eventually lead to pulp necrosis (1, 2). The pulp tissue has nutritive, protective, and formative functions and a healthy pulp facilitates normal functioning (3, 4). Therefore, the scientific communty is increasingly recognizing the advantages of vital pulp therapy (VPT) over traditional root canal treatment (5, 6). Pulp capping agents play a vital role in VPT. By covering the exposed pulp, these agents effectively reduce inflammatory responses, while also inducing migration and differentiation of deep and undamaged pulp stem cells, and promoting the formation of reparative dentin (7, 8). At present, as VPT is a research hotspot in the field, the search for new pulp-capping agents with high success rates and low trauma is a future trend in dental care.

Dexamethasone (DEX), a synthetic glucocorticoid, is widely used in various clinical departments, including endocrinology, oncology, and respiratory medicine (9–11). Furthermore, glucocorticoids exert analgesic effects by reducing the secretion of bradykinin and neuropeptides tissues, as well as inhibiting pain signaling in the noxious C-fibers (12, 13). The research focus on DEX has also extended to its role in promoting osteogenesis. A study showed that DEX stimulates the proliferation and differentiation of bone marrow mesenchymal stem into osteoblasts (14). Moreover, DEX has been found to enhance the expression of odontogenic differentiation-related genes such as dentin matrix protein 1 (DMP-1), Runt-related transcription factor 2 (Runx2), dentin sialophosphoprotein (DSPP), osteocalcin (OCN), and alkaline phosphatase (ALP), consequently improving the *in vitro* mineralization of human pulp cells (15). However, high concentrations of DEX inhibit osteogenesis. At a concentration of 10^-6^ mol/L, DEX disrupts mitochondrial dynamics, inhibits osteogenesis of stem cells, and promotes adipogenesis (16–18). Consequently, scientists have started investigating alternative mechanisms of topical drug delivery and strategies to increase its initial solubility without overloading the drug (19–21).

Hydroxyapatite (HA), a major component of natural bone, has no implant toxicity and is commonly used in bone repair due to its biocompatibility, bioactivity, osteoconductivity, osseointegration, and ability to induce osteogenesis under specific conditions (22, 23). Nano-HA, with a large surface area and excellent bioactivity, can stimulate the differentiation of dental pulp stem cells (DPSCs), thus promoting dentin regeneration and the formation of reparative dentin (24, 25). The spherical morphology and nanoscale nature of hollow hydroxyapatite (HHAM) offer distinct advantages over irregular HA, particularly in reducing physical damage *in vivo*. HHAM is well-suited for loading and releasing various drug molecules owing to its high surface area, low mass density, excellent properties for cell attachment, drug loading, and drug release kinetics (26–28). Thus, HHAM not only ensures localized and sustained drug release but also promotes bone healing (29, 30).

In this study, we aimed to assess the reparative potential of DEX-loaded hollow hydroxyapatite microspheres (DHHAM) as a direct pulp capping agent in a rat model of pulp injury. We hypothesized that DHHAM could effectively promote the formation of reparative dentin, promote the expression of DMP-1, and inhibit the expressions of tumor necrosis factor (TNF)-α, interleukin (IL)-6, and IL-1β.

## 2 Materials and methods

### 2.1 Preparation and *in vitro* release of DHHAM

A and B aqueous glycine solutions (Tianjin Guangfu Technology Development Co., Ltd., China) were prepared, and the concentration of glycine was adjusted to 20 mmol/L. Solution B was supplemented with a 15 mmol/L sodium dodecyl sulfate solution (Sinopharm Chemical Reagent Co., Ltd., China). Solutions A and B were then stirred in a water bath at 40°C for 30 min. Equal amounts of 1 mmol/L calcium chloride and sodium carbonate solutions (Tianjin Guangfu Technology Development Co., Ltd., China) were to solutions A and B, respectively, and stirring was continued for 30 min. Solution A was quickly poured into solution B and maintained at 40°C for 1 h. The mixture was filtered and dried to obtain calcium carbonate. A total of 0.5 g of calcium carbonate was added to 100 mL of distilled water. Next, 200 mL of 0.03 mol/L disodium hydrogen phosphate dodecahydrate (Tianjin Guangfu Technology Development Co., Ltd., China) was added to the suspension at a rate of 2 drops/3s while stirring. The reaction was carried out in a constant temperature water bath at 50°C for 2 h under normal pressure, and the pH was adjusted to 9–11 with 20% sodium hydroxide solution (Tianjin Guangfu Technology Development Co., Ltd., China). Subsequently, the suspension was filtered, and the filtrate was washed repeatedly with anhydrous ethanol and distilled water, and dried in a constant temperature drying oven at 80°C for 12 h to obtain HHAM.

Following the experimental method described by Zhang et al. (15), an 80 mg suspension of HHAM was prepared in 4 mL of DEX-ethanol solution (10 mg/mL). The suspension was sonicated for 20 min and slowly shaken in an oscillator at 37°C for 24 h. The suspension was then centrifuged at 4000 rpm/min for 5 min. The loading and encapsulation rates were calculated by measuring the absorbance of DEX in the supernatant at 240 nm using a UV spectrophotometer (SHIMADZU, Japan). The precipitate obtained after centrifugation was dried at 40°C for 24 h to obtain DHHAM.

DHHAM, 100 mg, was packed into a dialysis bag containing 3 mL of phosphate-buffered saline (PBS) solution. The bag was then sealed and placed in 20 mL of PBS solution. The solution outside the dialysis bag was collected in 0.5 mL aliquots at 0, 24,… up to 864 h. After each sampling, the collected volume was immediately replenished with an equal amount of PBS solution. The cumulative amount of DEX released and the release rates were determined at 240 nm using a UV spectrophotometer.

### 2.2 Establishing the pulp capping model

The animal experiments were approved by the Animal Management and Use Committee, School of Basic Medical Sciences, Jilin University (Approval number: 2022456). Sixty healthy male Wistar rats, 8-week-old, with complete permanent dentition and no dental caries, malocclusion, or periodontal disease, were purchased and acclimated for 10 days. The rats were randomly divided into 4 groups containing 5 rats each: Dycal, Control, HHAM, and DHHAM. Three-time points were set for the evaluation, namely 1, 2, and 4 weeks.

**Figure 1:**
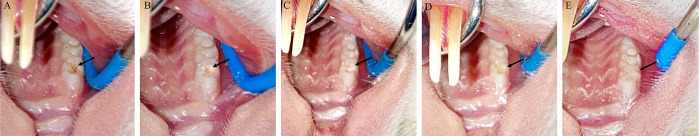
**(A)** Accidental pulp penetration **(B)** Placement of capping agent **(C)** Glass ionomer cushion **(D)** Fluid resin filling **(E)** Trimming and polishing after light curing.

The rats were anesthetized using isoflurane inhalation (Shanghai Yuyan Scientific Instruments Co., Ltd., China) followed by intraperitoneal injection of 3% pentobarbital sodium (1 mL/kg) (Merck, Germany). Next, the teeth were rinsed with saline. The tooth surface was then disinfected with 75% ethanol-soaked cotton. A cavity was then drilled on the proximal mesial surface of the left maxillary first molar using a 1/4 sterile ball bur (Dresdner Medical Instruments Co., Ltd., China). To prevent heat-induced pulpal damage, a new turning needle was used for each tooth, and the cavity was continuously rinsed with sterile distilled water. After slight reddening, the remaining dentin at the base of each cavity was penetrated using a sterile 15-gauge stainless steel c-file (Dentsply, USA) to reveal the pulp. To control bleeding, a sterile cotton ball was placed on the exposed area and pressed for 1–2 min. Once the bleeding was controlled, the perforations were directly covered with the respective material (5 µg/mL of HHAM, and DHHAM and Dycal prepared according to the instructions). In the Control group, the perforations were covered with saline. All the cavities were then filled with glass ionomer (Changshu Shang Dental Materials Co., Ltd., China) and liquid resin (3M, USA) for restoration according to the product description (**Figure 1**). The cusps of the teeth were slightly trimmed to reduce occlusal forces after light curing. A soft diet was administered after the procedure.

### 2.3 Animal sacrifice and specimen collection

Rats were anesthetized and sacrificed by cardiac perfusion fixation at 1, 2, and 4 weeks after the procedure. The left maxillary first molar and surrounding alveolar bone were isolated. The samples were processed and prepared according to the method described by Islam et al. (31). The samples were then sliced into 3 µm-thick sections in the mesial-distal direction using a sliding-type section cutter (Leica-2016, Germany). The slides were then numbered and subjected to staining using hematoxylin-eosin (H&E) for visualization. Next, immunohistochemical staining was conducted using the following primary antibodies: CD45 polyclonal (dilution 1:1000, Proteintech, USA), IL-6, DMP-1, TNF-α, and IL-1β (dilution 1:300, Affinity Bioscience, USA). After deparaffination in xylene, hydration was carried out using a series of graded alcohol solutions. The subsequent steps were performed following the manufacturer’s instructions for the immunohistochemical staining kit (Fuzhou Maixin Biotechnology Development Co., Ltd., China).

### 2.4 Histological analysis

Histological analysis was performed by a blinded observer using a light microscope and image capture system (Olympus, Japan) following image acquisition. The extent of reparative dentin formation was evaluated as the percentage of reparative dentin formed between the pulp perforation and the lowest point of the pulp chamber roof. The degree of inflammation was evaluated by determining the percentage of pulpal necrosis and inflammation in the area between the pulp perforation and the incision line. The incision line was on the most convex point of the pulp chamber wall in the proximal and distal mesial cusps of the section near the pulp perforation.

### 2.5 Statistical analysis

Statistical analyses were performed using analysis of variance (ANOVA). For multiple pairwise comparisons, Tukey’s multiple comparison tests were conducted. The statistical analysis was carried out using SPSS version 23.0. The threshold for statistical significance was set at p<0.05.

## 3 Results

### 3.1 Characterization of HHAM and cumulative release rate of DHHAM *in vitro*

**Figure 2:**
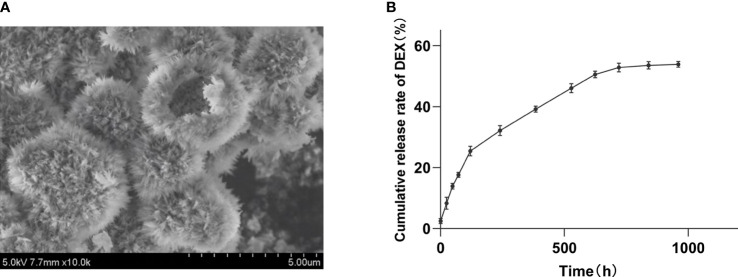
**(A)** DHHAM under FESEM; **(B)** Cumulative release rate of DEX from DHHAM.

HHAM was examined using field emission scanning electron microscopy (FESEM), revealing a diameter ranging from 2 to 4 µm. It was composed of numerous short, needle-shaped nanoparticles (**Figure 2A**). The encapsulation rate of DEX in HHAM was 34.6% and the drug loading rate was 14.3%. The 30-day *in vitro* sustained release cumulative release rate of DEX was 52.8%, and release rate and time showed a linear relationship (**Figure 2B**).

### 3.2 DHHAM reduces inflammatory responses of the dental pulp

#### 3.2.1 One week after surgery

**Figure 3:**
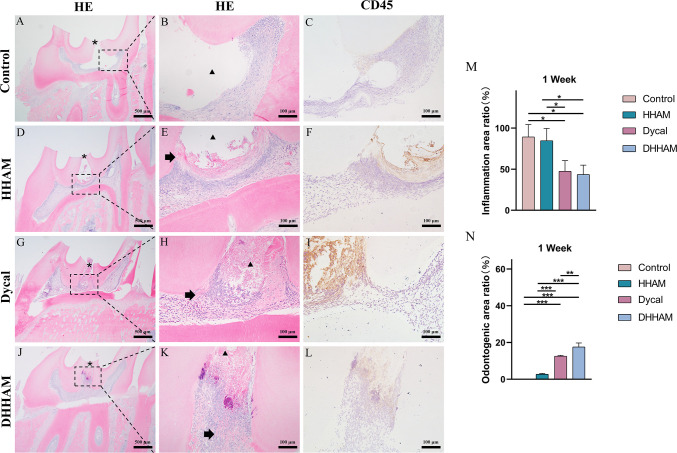
H&E and CD45 staining at 1 week. **(A, D, G, J)** (bar = 500 µm), **(C, F, I, L)** (bar = 100 µm); **(B, E, H, K)** are the magnified images of the rectangular area (bar = 100 µm). *= pulp perforation; ➔ = reparative dentin; ▴= pulp necrosis. **(M)**: Statistical chart of the percentage of inflamed area at 1 week (n=3); **(N)**: statistical chart of the percentage of reparative dentin area formed at 1week (n=3). *p<0.001; **p<0.01; ***p<0.05.

**Figure 4:**
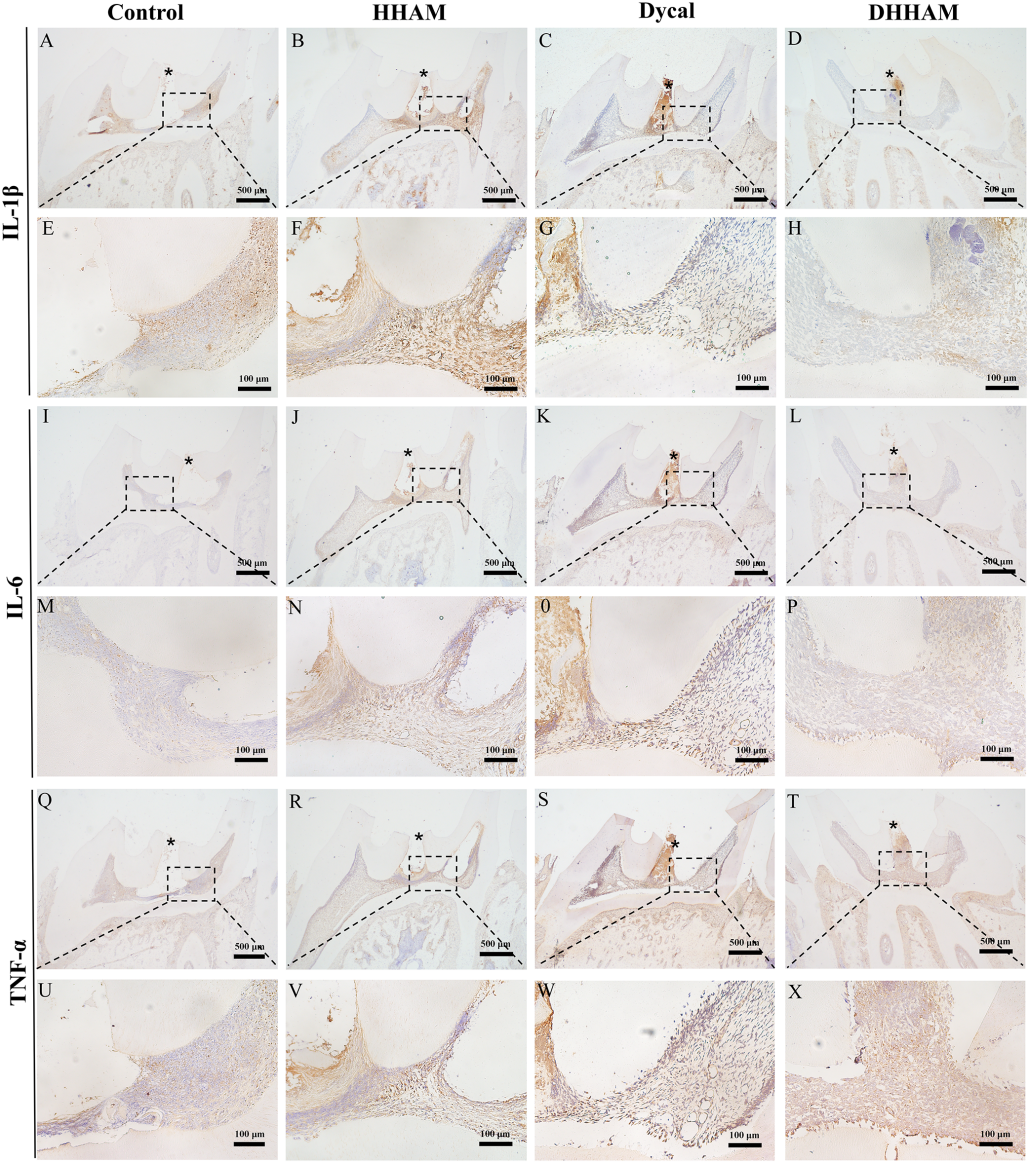
Immunohistochemical staining of IL-6, TNF-α, and IL-1β at 1 week. **(A–D, I–L, Q–T)** (bar = 500μm); **(E–H, M–P, U–X)** are the magnified images of the rectangular area (bar = 100 μm). *= pulp perforation.

In both the Control and HHAM groups, a majority of the inflammatory cells infiltrated 1/3rd of the coronary pulp, resulting in partial necrosis of the pulp. However, the inflammation in the Dycal and DHHAM groups was mild and restricted to the area below the drug contact zone (**Figure 3**). The H&E and CD45 staining showed that the degree of inflammation in the DHHAM group was significantly lower compared to the Control and HHAM groups (p<0.05). The immunohistochemical staining revealed significantly high expression levels of IL-6, TNF-α, and IL-1β in the odontoblasts, fibroblasts, and inflammatory cells throughout the coronary medulla in each group, and the strongest expression was in the area near the pulp perforation (**Figure 4**). However, the expression levels of these three antibodies in the Dycal and DHHAM groups were lower compared to the other two groups. Additionally, the expression areas in the DHHAM and Dycal groups were smaller in comparison to the Control and HHAM groups.

#### 3.2.2 Two weeks after surgery

**Figure 5:**
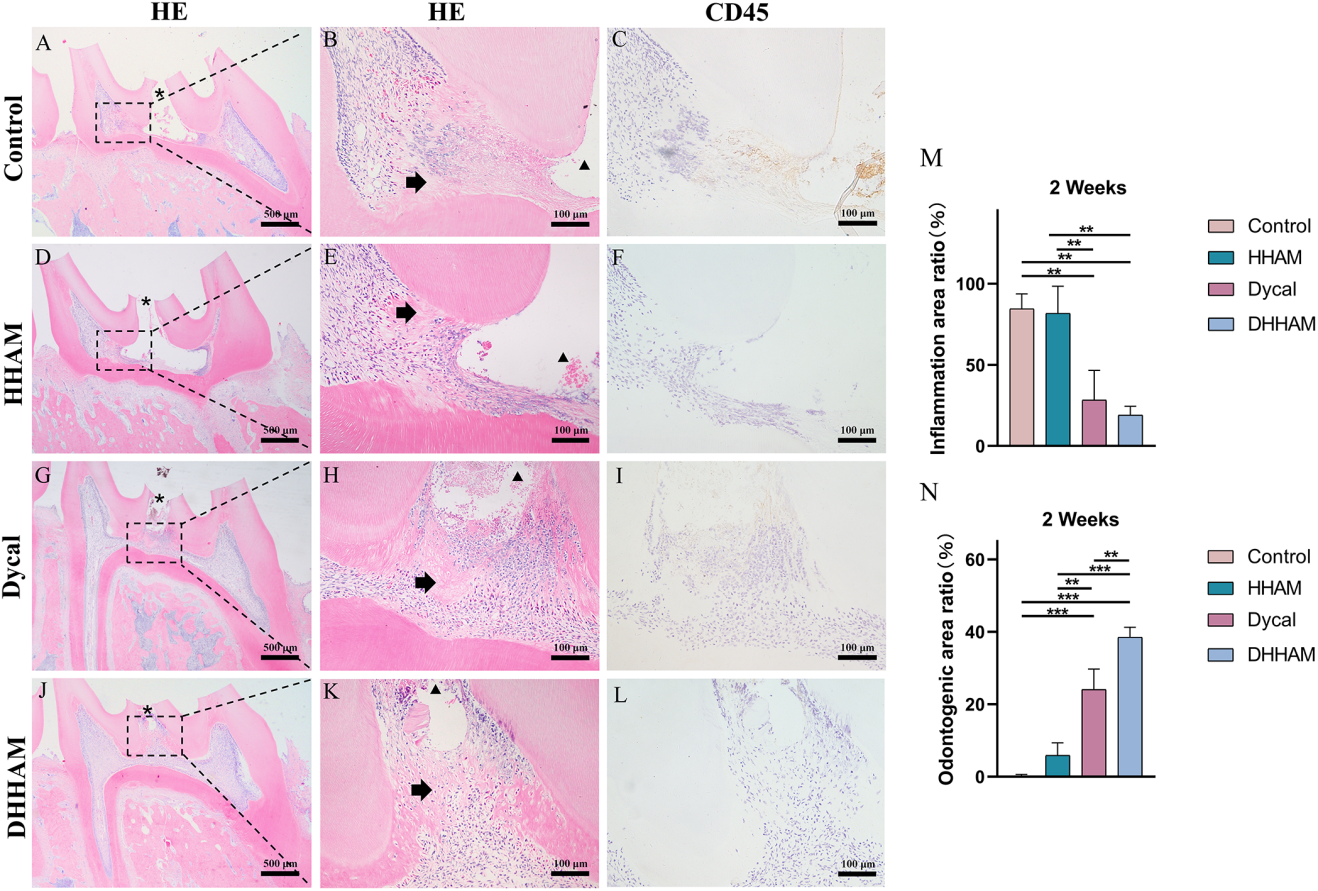
H&E and CD45 staining at 2 weeks. **(A, D, G, J)** (bar = 500 µm), **(C, F, I, L)** (bar = 100 µm); **(B, E, H, K)** are the magnified images of the rectangular area (bar = 100 µm). *= pulp perforation; ➔ = reparative dentin; ▴= pulp necrosis. **(M)**: Statistical chart of the percentage of inflamed area at 2 weeks (n=3); **(N)**: statistical chart of the percentage of reparative dentin area formed at 2 weeks (n=3). *p<0.001; **p<0.01; ***p<0.05.

**Figure 6:**
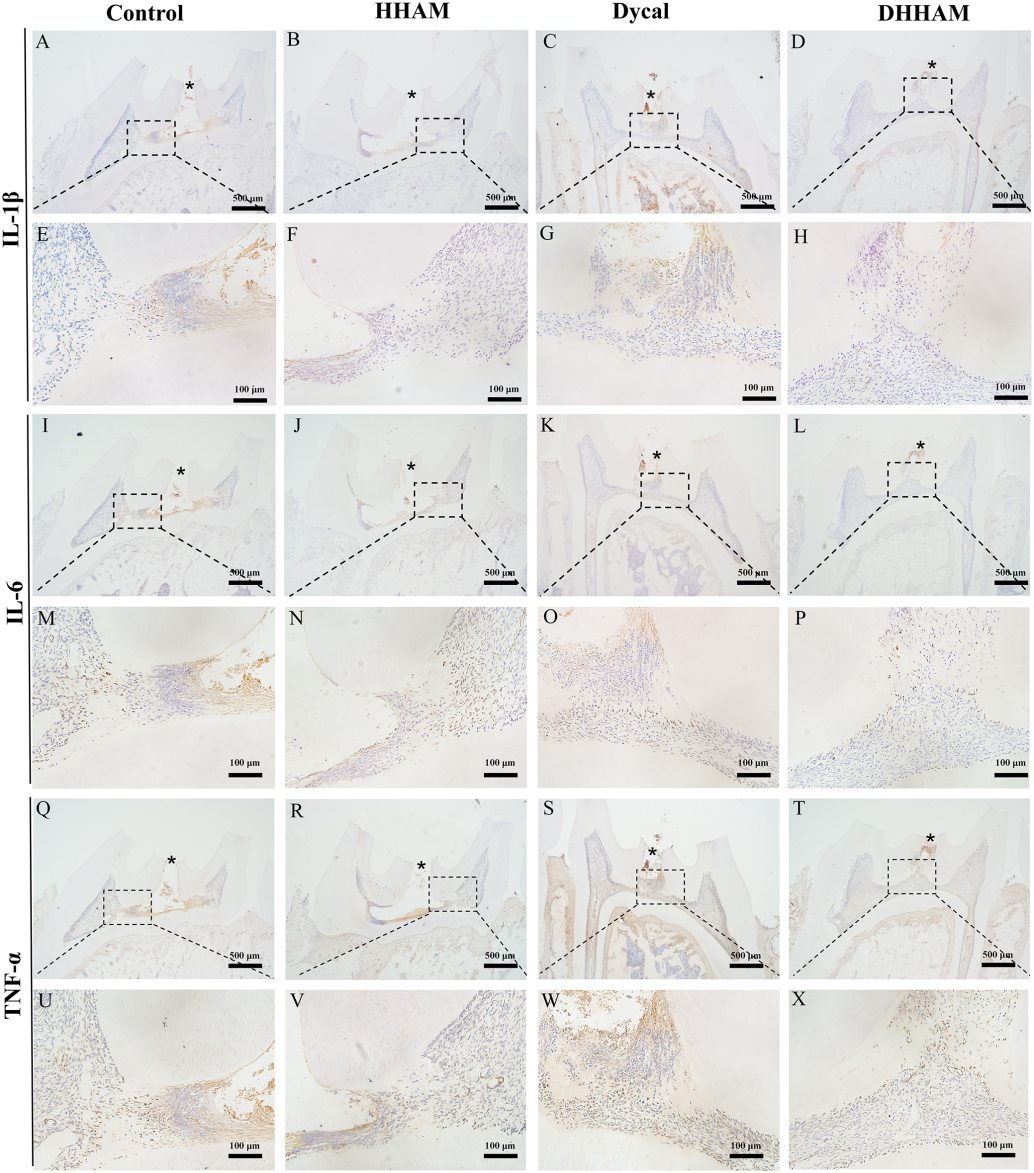
Immunohistochemical staining of IL-6, TNF-α, and IL-1β at 2 weeks. **(A-D, I–L, Q–T)** (bar = 500μm); **(E–H, M–P, U–X)** are the magnified images of the rectangular area (bar = 100 μm). *= pulp perforation.

The samples in the Control and HHAM groups showed severe pulp inflammation, with a majority of inflammatory cells infiltrating more than 1/3rd of the coronal pulp, leading to partial pulp necrosis and abscess formation. The majority of inflammatory cells in the Dycal group infiltrated 1/3rd of the coronal pulp, and the pulp vessels were dilated and congested (**Figure 5**). Conversely, samples in the DHHAM group showed only mild inflammation below the drug contact area, with fewer inflammatory cells and reduced inflammation relative to the Control and HHAM groups (p<0.01). Notably, the expression levels of IL-6, TNF-α, and IL-1β were low in all groups relative to those in week 1. IL-1β was expressed in inflammatory cells, odontoblasts, and some fibroblasts, and covered almost the whole coronal pulp in the Control group. In the HHAM group, IL-1β was expressed in approximately 1/3rd of the coronal pulp. However, IL-1β was only expressed in a small number of odontoblasts and fibroblasts in the Dycal and DHHAM groups and was weaker in intensity than the other two groups (**Figure 6**). IL-6 was highly expressed in inflammatory cells, odontoblasts, and some fibroblasts in approximately 2/3rd of the coronal pulp in the Control and HHAM groups. In the Dycal group, IL-6 was mainly expressed in inflammatory cells, odontoblasts, and fibroblasts in 1/3rd of coronal pulp. In the DHHAM group, it was only expressed in a small number of odontoblasts and fibroblasts, and this range was smaller than those of the other three groups (**Figure 6**). TNF-α was expressed in approximately 2/3rd of the coronal pulp in the Control and HHAM groups and was expressed mainly in inflammatory cells, odontoblasts, and fibroblasts in 1/3rd of the coronal pulp in the Dycal group. In the DHHAM group, TNF-α was expressed only in a small number of odontoblasts and fibroblasts, and the expression levels were lower than those in the Control and HHAM groups (**Figure 6**).

#### 3.2.3 Four weeks after surgery

**Figure 7:**
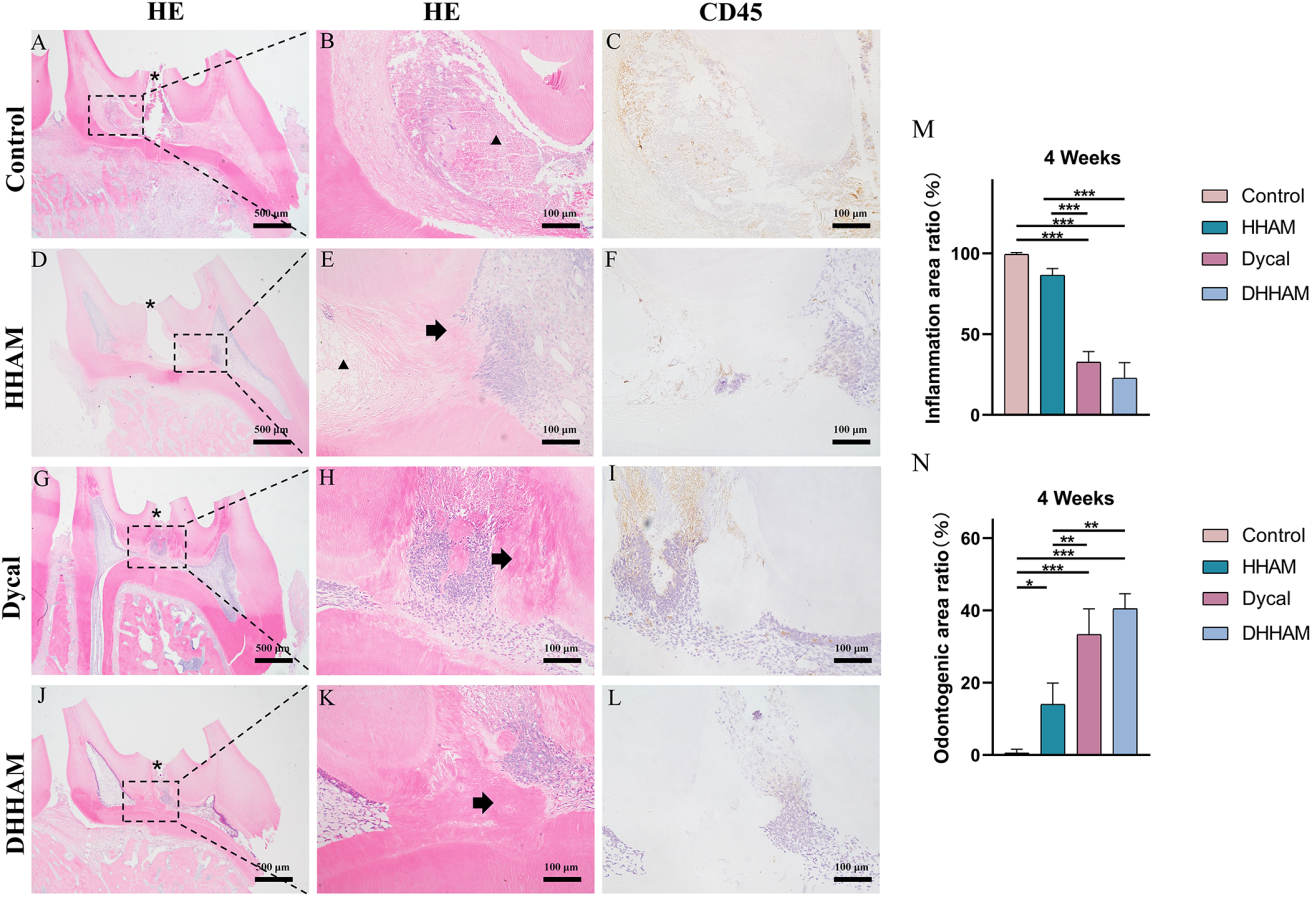
H&E and CD45 staining at 4 weeks. **(A, D, G, J)** (bar = 500 µm), **(C, F, I, L)** (bar = 100 µm); **(B, E, H, K)** are the magnified images of the rectangular area (bar = 100 µm). *= pulp perforation; ➔ = reparative dentin; ▴= pulp necrosis. **(M)**: Statistical chart of the percentage of inflamed area at 4 weeks (n=3); **(N)**: statistical chart of the percentage of reparative dentin area formed at 4 weeks (n=3). *p<0.001; **p<0.01; ***p<0.05.

**Figure 8:**
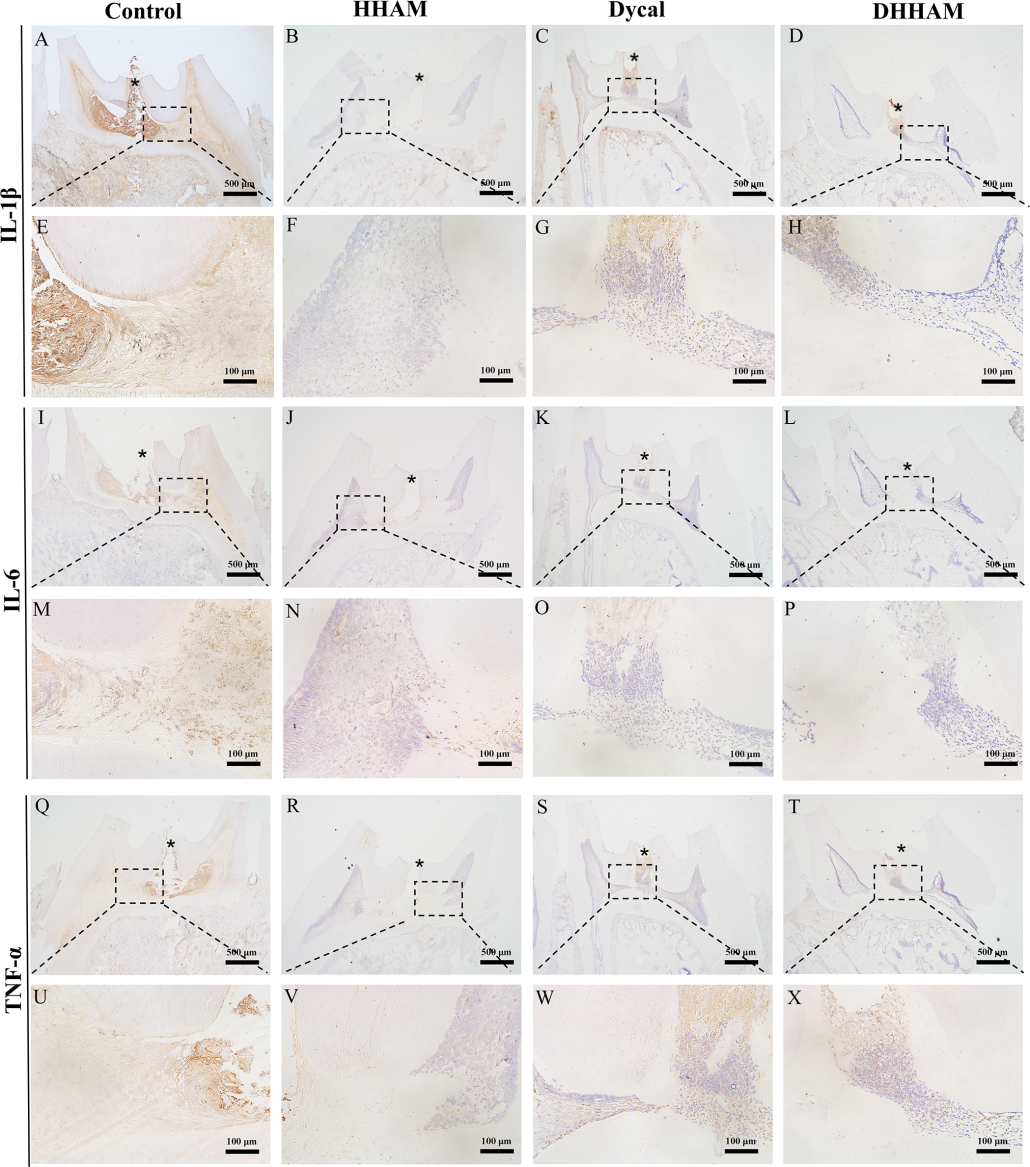
Immunohistochemical staining of IL-6, TNF-α, and IL-1β at 4 weeks. **(A–D, I–L, Q–T)** (bar = 500μm); **(E–H, M–P, U–X)** are the magnified images of the rectangular area (bar = 100 μm). *= pulp perforation.

Samples in the Control group exhibited almost complete necrosis of the coronary pulp. In the HHAM group, inflammation was predominantly observed in 2/3rd of the crown pulp, with noticeable pulp vessel dilation. However, few inflammatory cells were present in samples in the Dycal group, with most of the inflammation occurring below the drug contact area (**Figure 7**). The inflammatory manifestations in the DHHAM group were similar to those in the Dycal group, and the degree of inflammation was significantly lower compared to the Control and HHAM groups (p<0.001). In the Control group, IL-6, TNF-α, and IL-1β were expressed in inflammatory cells and some fibroblasts in the whole dental pulp. In contrast, in the Dycal and the DHHAM group, IL-6, TNF-α, and IL-1β were mainly expressed in inflammatory cells, odontoblasts, and fibroblasts in the coronal pulp, with the highest expression in and around the reparative dentin, and this expression level was lower compared to those in the other two groups (**Figure 8**).

### 3.3 DHHAM promotes the formation of reparative dentin

#### 3.3.1 One week after surgery

**Figure 9:**
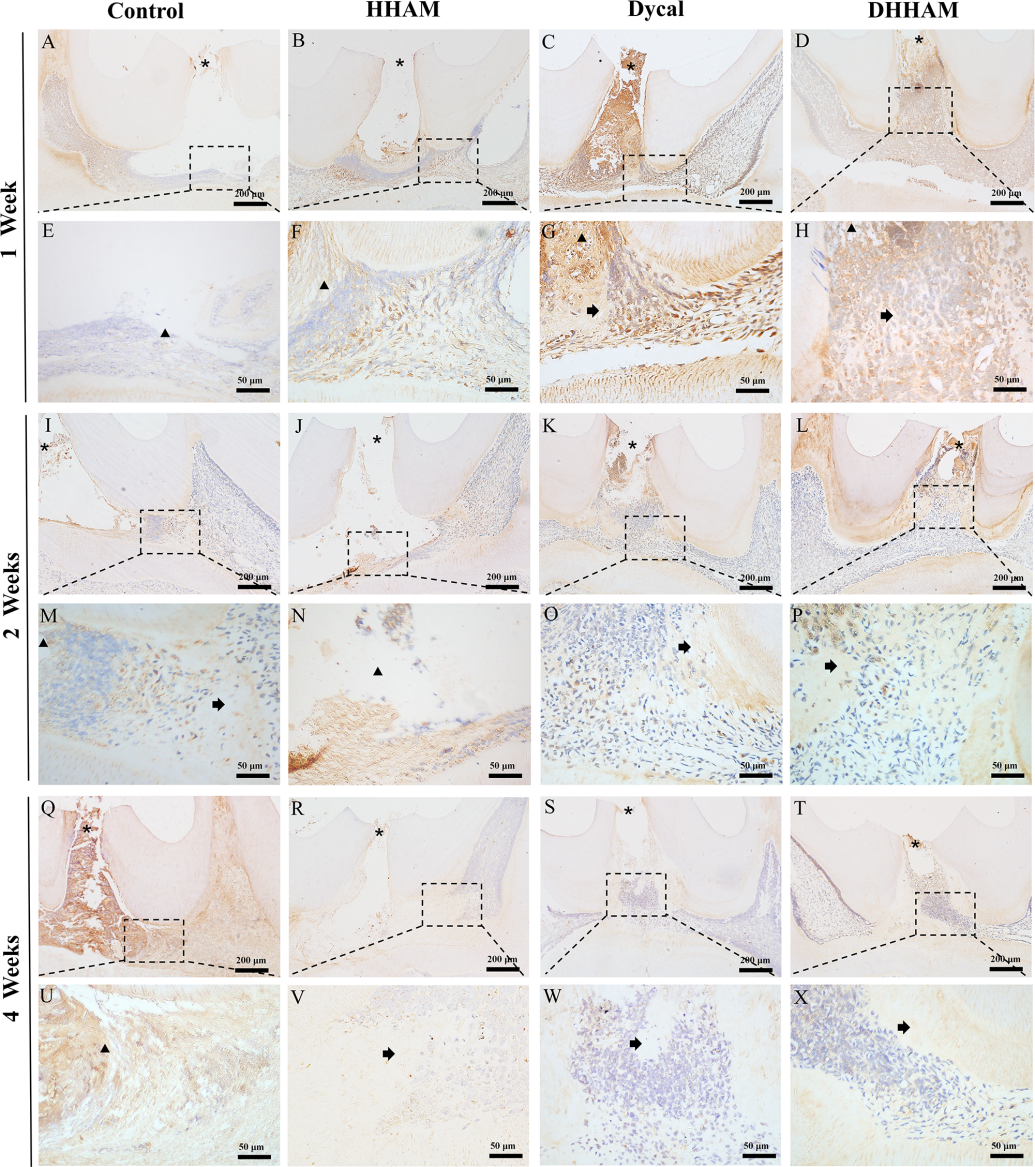
Immunohistochemical staining of DMP-1 at 1, 2, and 4 weeks. **(A–D, I–L, Q–T)** (bar = 200 mm); **(E–H, M–P, U–X)** are the magnified images of the rectangular area (bar = 50 mm). *= pulp perforation; ➔ = reparative dentin; ▴= pulp necrosis.

No significant reparative dentin formation was observed in the Control group. In the HHAM group, scattered mineralized tissue was observed around the necrotic area in the majority of samples. Samples in the Dycal and DHHAM groups exhibited calcified masses below the pulp perforation (**Figure 3**). Significant differences were observed in the percentage of reparative dentin formation, with the DHHAM group displaying a higher percentage compared to the Control, HHAM (p<0.001), and Dycal groups (**Figure 3N**). In the Control, HHAM, and Dycal groups, DMP-1 expression was detected around the inflammatory area. However, in the DHHAM group, DMP-1 expression was detected in odontoblasts and fibroblasts below the pulp perforation, and partial formation of the mineralized matrix was observed (**Figure 9**).

#### 3.3.2 Two weeks after surgery

Reparative dentin formation was observed in all samples in the Control and HHAM groups, limited to the region between the apex and base of the pulp chamber. In the Dycal group, incomplete dentin bridges were observed below the pulp perforations in all samples, with the presence of odontoblasts further below (**Figure 5**). The DHHAM group exhibited a significantly higher percentage of reparative dentin formation compared to the Control, HHAM (p<0.001), and Dycal (p<0.01) groups (**Figure 5N**). In the Control and HHAM groups, DMP-1 was considerably expressed around the areas of necrosis and inflammation, accompanied by matrix mineralization. In the DHHAM group, considerably high expression levels of odontoblasts and fibroblasts were detected under the perforated hole, along with the formation of a large amount of mineralized matrix. The Dycal group showed similar results as the DHHAM group, but the area was comparatively smaller in the Dycal group (**Figure 9**).

#### 3.3.3 Four weeks after surgery

Reparative dentin formation was observed in all samples in the Control and HHAM groups, limited to the region between the apex and base of the pulp chamber. The quality of reparative dentin in the HHAM group was found to be superior to that in the Control group. In the Dycal group, incomplete thick dentin bridges were formed below the perforation cavity in all samples. Similarly, in the DHHAM group, incomplete dentin bridges were observed below the perforations, effectively closing them, and large areas of reparative dentin were visible in the crown pulp (**Figure 7**). The DHHAM group exhibited a significantly higher percentage of reparative dentin compared to the Control (p<0.001) and HHAM groups (p<0.01). However, there was no statistically significant difference between the DHHAM and Dycal groups (p>0.05) (**Figure 7N**). In the Control group, the majority of samples displayed enlarged necrotic areas with low expression levels of DMP-1 around these areas. In the HHAM group, low expression levels of DMP-1 were detected around the necrotic and inflammatory areas. The majority of the samples showed low expression levels, indicating that the pulp had largely returned to its normal state (**Figure 9**).

## 4 Discussion

The preservation of the living pulp after a pulpal injury is a controversial issue. Dental caries and traumatic lesions can irreversibly damage the pulp and induce excessive inflammatory responses that can be detrimental to pulp repair. Therefore, it is crucial to promptly treat inflammation and enhance the self-healing capacity of pulp resident cells. Calcium hydroxide is regarded as the gold standard of traditional materials used in PVT (32). Unfortunately, calcium hydroxide is soluble and prone to dentin bridge formation with tunnel-like defects. These defects provide an invasive pathway for microorganisms, ultimately leading to treatment failure in preserving the living pulp (33–35). Consequently, there is ongoing research to identify new direct pulp-capping materials that can stimulate pulp regeneration. Previous *in vitro* studies have demonstrated that DHHAM promotes the odontogenic differentiation of human dental pulp cells (15). This study examined the effect of DHHAM on inflammation and dentin regeneration in injured pulp tissue.

Glucocorticoids are the main stress hormones that regulate physiological processes (36). DEX, a glucocorticoid, has potent anti-inflammatory and analgesic effects. It has several applications in root canal treatment, including anesthesia, oral administration, and root canal irrigation (37–41). DEX also activates the classical Wnt signaling pathway to enhance osteoblast growth and differentiation (42). On the other hand, HA increases local Ca^2+^ concentration, thereby activating osteoblast proliferation and promoting the differentiation of mesenchymal stem cells. Additionally, HA exhibits excellent performance in bone repair, bone replacement, drug delivery, and slow release due to its non-immunogenicity, biocompatibility, and favorable osteoconductivity (23, 43). In this experiment, we incorporated DEX into HHAM and found that DHHAM had a linear *in vitro* release profile without any significant abrupt release, indicating the successful development of a controlled drug delivery system. Notably, long-term or high-dose use of glucocorticoids is associated with side effects such as hypertension, osteoporosis, obesity, and peptic ulcer disease (44–46). To avoid these side effects, this study developed a sustained release system that ensures a sustained low-dose release of DEX administered in a single local dose (47).

The *in vitro* studies revealed that DHHAM at a concentration of 1 µg/mL yielded the most favorable results in promoting the differentiation of dental pulp cells. We also performed ALP staining, alizarin red staining, and quantitative polymerase chain reaction at this concentration. The results showed that DHHAM improved the *in vitro* mineralization of human dental pulp cells and upregulated the expression of dental genes such as DMP-1 and ALP, which promoted the dental differentiation of dental pulp stem cells (15). To further explore the potential efficacy of DHHAM as a pulp capping material, we conducted pre-experiments using different concentrations of DHHAM, namely 1, 3, and 5 µg/mL. The results showed that 5 µg/mL DHHAM had the most favorable effect on pulp capping. Consequently, this concentration was chosen for subsequent animal experiments.

Animals used for capped pulp studies include monkeys, dogs, ferrets, miniature pigs, rabbits, rats, and mice. Rat molars are commonly used due to their similarities to human molars in terms of anatomy, histology, biology, and physiology. Additionally, using rat molars offers advantages such as lower cost, simplicity of operative procedures, ease of feeding, and high survival rates (48, 49). Therefore, we constructed the animal model of capped pulp using rat molars. To create the pulp exposure in the rat molars, we used sterile C-files. This approach not only prevented damage to the pulp caused by rotating needles during the cutting process but also ensured consistency in the size of the pulp perforation cavity.

CD45 is widely expressed in inflammatory cells such as lymphocytes and microglia and is a key molecule in cell signaling processes (50). It also plays an essential role in lymphocyte development and maturation and functional regulation. In addition, CD45 is an inflammatory cell marker for assessing tissue inflammation (50). Therefore, we examined the extent of pulp necrosis and inflammation using H&E and CD45 staining. The DHHAM group exhibited lower levels of inflammation compared to the HHAM and Control groups at 1, 2, and 4 weeks, indicating that DEX had an excellent anti-inflammatory effect. These findings are consistent with the conclusions of previous research (51). The anti-inflammatory effects of DEX may be related to its ability to induce lymphocyte apoptosis, inhibit pro-inflammatory transcription factor release, and stimulate the expression of anti-inflammatory genes (52). Extensive evidence supports the binding of glucocorticoids to the glucocorticoid receptor (GR), followed by translocation from the cytoplasm to the nucleus. Once in the nucleus, the glucocorticoid-GR complex binds to the glucocorticoid response element present in the promoter region of target genes, leading to the transactivation of the transcription of anti-inflammatory genes such as Annexin A1 (AnxA1) (53). Glucocorticoid activation of AnxA1 inhibits cytoplasmic phospholipase A2, thereby preventing the synthesis of eicosanoids such as prostaglandins and leukotrienes. In addition, glucocorticoid activation inhibits the expression of cyclooxygenase 2, thereby inhibiting the release of pro-inflammatory mediators and inhibiting leukocyte migration by modulating the interaction between endothelial cells and leukocyte surface adhesion molecules (54). Glucocorticoids can also exert anti-inflammatory effects by trans-suppressing the expression of pro-inflammatory transcription factors, including activating protein-1 (AP-1) and nuclear factor-kappa B (NF-κB). Glucocorticoids inhibit AP-1 activity by directly interfering with c-Jun-mediated transcription and inducing mitogen-activated protein kinase phosphatase 1 (52). Glucocorticoids also indirectly inhibit NF-κB transcription by directly inhibiting NF-κB or inducing leucine zip expression, thereby inhibiting the expression of chemokines, TNF-α, cell adhesion molecules, IL-1β, matrix metalloproteinases, and IL-6 (55, 56).

In this experiment, we observed that the expression levels of IL-6 and IL-1β in the DHHAM group were consistently lower than those in the Control and HHAM groups at all examined time points. IL-1β is known to have multiple functions in the immune system. It can recruit macrophages, activate other pro-inflammatory cytokines such as IL-8 and IL-6, and regulate the expression of chemokines (57). Moreover, IL-1β induces inflammatory responses in almost all tissues and organs and is considered a key mediator in promoting various innate immune processes and exacerbating damage during acute tissue injury and in chronic diseases (58, 59). Hence, inhibiting IL-1β may reduce cellular injury in inflammation. IL-6, considered a key regulator of chronic and acute inflammation and a stimulating factor for B and T cells, induces lymphocytes, macrophages, and neutrophils during inflammation. Notably, excessive production of IL-6 has been implicated in the pathogenesis of various inflammatory diseases (60). Therefore, we hypothesized that DHHAM could reduce inflammatory responses by inhibiting the expressions of IL-6 and IL-1β.

TNF-α is another critical pro-inflammatory cytokine with pleiotropic functions, and clinical evidence shows elevated levels of TNF-α in the inflamed dental pulp (60, 61). Therefore, a decrease in TNF-α levels may contribute to a reduction of pulp inflammation. Our results showed that the intensity and range of TNF-α expression were less than those in the Control and HHAM groups at 2 and 4 weeks, indicating that DHHAM could reduce inflammatory responses by suppressing the expression of TNF-α. However, the intensity and range of TNF-α expression at 1 week were not significantly different from those of the other groups. We speculate that this may be attributed to the relatively weaker effect of DEX in inhibiting TNF-α expression in pulp inflammation, as compared to its effects on IL-1β and IL-6, which is consistent with the findings of Lang et al. (62).

We observed reparative dentin formation by H&E staining and found that the percentage of reparative dentin formation was markedly higher in the DHHAM than in the HHAM and Control groups after 1, 2, and 4 weeks of pulp capping, indicating that DEX promotes dentin formation. These results are consistent with those published by Hayashi et al. (63) and Okamoto et al. (64). In addition, the percentage of reparative dentin formation at 4 weeks was higher in the HHAM group than in the Control group, indicating that HHAM promoted dentinogenesis (23). The percentage of reparative dentin formation was higher in the DHHAM group than in the HHAM group, which further demonstrated the anti-inflammatory and pro-dentinogenic effects of DEX. Although the percentage of dentin formation was higher in the DHHAM group than in the Dycal group at 1 and 2 weeks, there was no statistically significant difference at 4 weeks, suggesting that DHHAM promoted the formation of reparative dentin earlier than Dycal. The results are consistent with those of Alagha et al. (65).

DMP-1, a biomarker for assessing the activity of adult dental cells, plays a role in regulating HA nucleation by binding to Ca^2+^ ions (25, 66). In this study, significantly high expression levels of DMP-1 were observed in the DHHAM group at 1 and 2 weeks, suggesting that DHHAM can stimulate the proliferation and migration of stem cells toward the exposed pulp surface, promoting their differentiation into odontoblasts (31). At 4 weeks, the DMP-1 expression levels decreased, and thicker dentin bridges were observed, indicating that the pulp had largely returned to its normal state.

The success of VPT depends on rigorous case selection; however, assessing the true pulp status in the clinical setting is challenging and crucial for assessing prognosis (67). The present study established a model of accidental pulp penetration in the pulp of healthy rats; accidental pulp exposure has a better prognosis compared to pulp exposure of carious origin. Therefore, the indications for clinical application need to be carefully considered. Moreover, this experiment represents a preliminary study on the role of DHHAM in VPT, and a larger sample size, longer observation time, and related mechanistic studies are needed to further explore the role of DHHAM in VPT.

## 5 Conclusion

These findings demonstrated that DHHAM effectively promoted the early formation of reparative dentin in the rat accidental pulp penetration model, surpassing the effects of Dycal. DHHAM also promoted dentin mineralization by promoting the expression of DMP-1 and inhibited IL-6, TNF-α, and IL-1β, resulting in an excellent anti-inflammatory effect. These findings present a promising avenue for the development of a living pulp preservation strategy.

## Data availability statement

The raw data supporting the conclusions of this article will be made available by the authors, without undue reservation.

## Ethics statement

The animal study was reviewed and approved by the Animal Management and Use Committee, School of Basic Medical Sciences, Jilin University.

## Author contributions

XL designed the study. XL, YX and LNZ acquired the data. WG, LH and LJZ analyzed and interpreted the data. XL wrote the paper. YL critically revised the manuscript for important intellectual content. YL provided financial support. All authors contributed to the article and approved the submitted version.
